# Bioprospecting on invasive plant species to prevent seed dispersal

**DOI:** 10.1038/s41598-017-14183-5

**Published:** 2017-10-23

**Authors:** Lorenzo Guzzetti, Andrea Galimberti, Ilaria Bruni, Chiara Magoni, Maura Ferri, Annalisa Tassoni, Enrico Sangiovanni, Mario Dell’Agli, Massimo Labra

**Affiliations:** 1 Zooplantlab, Department of Biotechnology and Biosciences, University of Milano-Bicocca, Piazza della Scienza 2, I-20126 Milano, Italy; 2 Department of Biological, Geological and Environmental Sciences, University of Bologna, via Irnerio 42, 40126 Bologna, Italy; 3 Department of Civil, Chemical, Environmental and Materials Engineering, University of Bologna, via Terracini 28, 40131 Bologna, Italy; 4 Laboratory of Pharmacognosy, Department of Pharmacological and Biomolecular Sciences, Università degli Studi di Milano, Via Balzaretti 9, 20133 Milano, Italy

## Abstract

The most anthropized regions of the world are characterized by an impressive abundance of invasive plants, which alter local biodiversity and ecosystem services. An alternative strategy to manage these species could be based on the exploitation of their fruits in a framework of bioprospecting to obtain high-added value compounds or phytocomplexes that are useful for humans. Here we tested this hypothesis on three invasive plants (*Lonicera japonica* Thunb., *Phytolacca americana* L., and *Prunus serotina* Ehrh.) in the Po plain (northern Italy) which bear fruits that are highly consumed by frugivorous birds and therefore dispersed over large distances. Our biochemical analyses revealed that unripe fruit shows high antioxidant properties due to the presence of several classes of polyphenols, which have a high benchmark value on the market. Fruit collection for phytochemical extraction could really prevent seed dispersal mediated by frugivorous animals and produce economic gains to support local management actions.

## Introduction

Biological invasions constitute a global environmental, economic, and social change driver as they can irremediably alter ecosystem compositions and services, thus determining a loss in terms of biodiversity, agriculture productivity, and human health^1,2^. In Europe, the number of invasive species has been rapidly increasing and reached an estimated value of over 12000 species including plants, animals, fungi, and microorganisms in 2016^3^. Concerning plants, their intentional introduction for ornamental purposes is a major pathway for invasion^4^. The effect of invasive plants on natural ecosystems can be disastrous, firstly for the competition with indigenous species^5^, and secondly, because they limit the stability and availability of natural resources to the whole native species community^6,7^. Detrimental effects on human health mediated by invasive plants have also been documented^2^. These include the occurrence of immunostimulant substances or allergenic factors, such as those on the pollen of some species or toxic metabolites in fruits, leaves, or other plant portions^8^. A typical example is *Ambrosia artemisiifolia* L. and *A*. *trifida* L. which pollens induce respiratory diseases, and management costs to avoid allergy outbreaks are very high^9^.

In the attempt to contrast the risks posed by alien and invasive plant species, management actions have been implemented to prevent introductions^4^, eradicate infestations before they become established^10,11^, and contain the spread of abundant or resistant taxa^4,12–14^. In many cases, management strategies (e.g., cutting, mowing and extirpation) are not completely effective, either for eradication or containment purposes, because invasive species rely on efficient dispersal systems ensuring their long-term persistence. Moreover, these strategies demand huge economic resources, not only to assist direct containment, but also for the management of the biomass resulting from extirpation and cutting activities^15^.

Considering that climate change is likely to exacerbate the negative effects of invasive alien species, as it will foster their further spread especially in the most fertile areas^9^, the economic resources and human energy demanded for managing invasive plants are expected to increase. The engagement of the citizen and other stakeholders to improve actions aimed at controlling the invaders creates societal challenges that policy makers are asked to prioritize^16^. At the same time, identifying innovative ways to transform invasive plants from ‘problems to resources’ also represent an important challenge for modern societies. In this context, we propose a bioprospecting strategy to find new compounds and antioxidant phytocomplexes to be used in different sectors of human interest (e.g. pharmaceutical, cosmetic, and nutrition). Usually, bioprospecting activities involve wild species from unexplored forests^17^, however, invasive species could also hide interesting chemical profiles or biological activities.

The technical goal of this study was to explore the chemical and biological properties of fruit from three invasive plants; *Lonicera japonica* Thunb., *Phytolacca americana* L., and *Prunus serotina* Ehrh. These species produce a large amount of fleshy fruits that are rich in secondary metabolites. Moreover, seeds of these plants represent the main tool for plant invasion and therefore, their collection and disposal for bioprospecting aims, could represent a suitable and innovative strategy for containment purposes.

**Figure 1:**
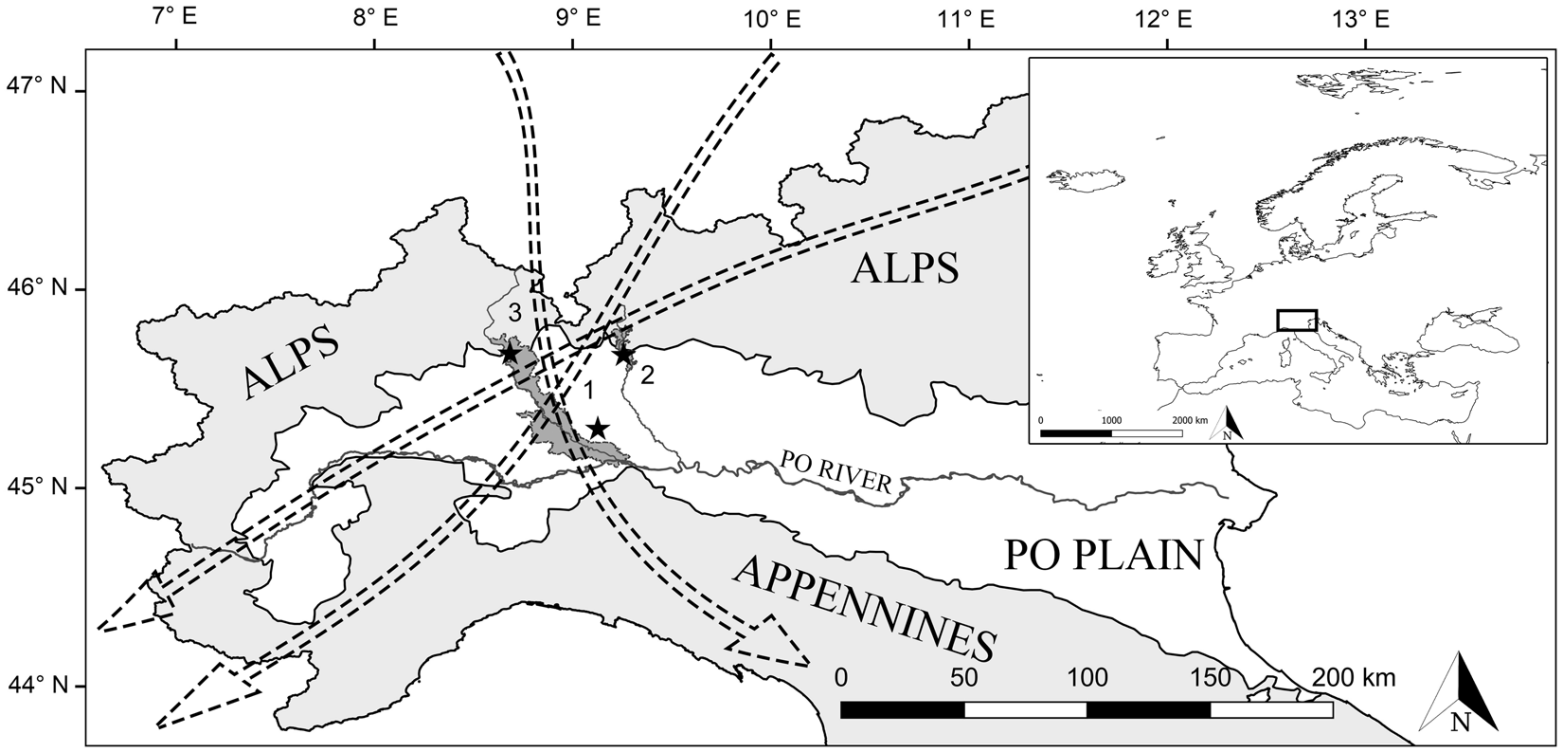
Study area. Location of the Italian Po Plain in the Western Palearctic region (upper right box) and sampling sites (1: Cassinazza di Baselica; 2: Valle Lambro Park; 3: Ticino Park). Mountain chains are indicated in light gray, whereas the parks investigated in this study are indicated in dark gray. Arrows represent some of the main bird migration routes crossing the Po plain region. The map has been generated using QGis desktop 2.18.3 and modified with Adobe Photoshop CC. The source map was downloaded from the public dataset (http://thematicmapping.org/downloads/world_borders.php) licensed under a Creative Commons Attribution-ShareAlike 3.0 Unported License (https://creativecommons.org/licenses/by-sa/3.0/).

All the three selected invasive species are very abundant in the Po plain region of Northern Italy. Like other European floodplains, this geographical area is increasingly colonized by invasive species due to the huge amount of water availability, fertile soils, and suitable climatic conditions^18^. The Po plain is characterized by high human disturbance resulting in a consistent alteration of local floral communities and an increasing rate of habitat fragmentation; these conditions support the process of invasion^19^. In addition, the Po plain is crossed by important migration routes (Fig. 1) and contains many refueling stopover sites for migratory birds species crossing the Alpine barrier to reach breeding sites farther north in spring and wintering grounds farther south in autumn^20^. Considering that the fruit from *L*. *japonica*, *P*. *americana*, and *P*. *serotina* are highly appreciated by frugivorous bird species, these last represent the primary vector for the diffusion and settlement of fleshy-fruiting plants^20–22^. For these reasons, the selection of efficient management activities to contrast the diffusion of invasive plant species represents a priority issue, not only to preserve local biodiversity but also to prevent the spread of these species in bird stopover areas along transcontinental migratory routes.

The rationale of the present study is to attract the general public and industry (i.e. pharmaceutical, cosmetic, and food) attention on these plants and their fruits in order to induce a massive exploitation for the extraction and valorization of their phytochemicals, which have beneficial effects on human health. This strategy could contribute to preventing the zoochorous dispersal of invasive seeds, while also producing economic benefits to support biodiversity management.

## Methods

### Study area and investigated plant species

The study area selected for sampling invasive plants was in the Lombardy Region and specifically, the natural areas of the Po plain (Fig. 1).

Parks and reserves of this floodplain are full of invasive plants (i.e., about 619 aliens out of 3820 plant recorded species)^19^ and include a lot of suitable stopover sites exploited by millions of frugivorous birds crossing the Alps during fall migration. These areas are also subjected to a progressive erosion of local plant diversity due to the consistent spread of invasive species^20^. Among alien taxa inhabiting the Po plain, *L*. *japonica*, *P*. *americana*, and *P*. *serotina* represent the most invasive species.

*L*. *japonica* is a Japanese plant introduced in Europe for ornamental purposes during the 19th century. In the Po plain, this species colonized different habitats, particularly mesophyll degraded woods and floodplains. This species is very attractive to birds, which feed on its fruit and contribute to its dispersal^23^. It produces about 90 seeds per plant and it is able to propagate by exploiting an efficient vegetative reproductive strategy and reaching sexual maturity within one year after settlement^24^. For these reasons, this species is included in the Black List of exotic invasive species in Lombardy^19^.

*P*. *americana* is another North American species introduced in Europe since the 16th century and is considered by some authors as naturalized in many European countries^19^. Nevertheless, it outcompetes local flora, since its numerous seeds (i.e. about 1500 seeds per plant) are scattered by a wide panel of frugivorous mammals and birds^25^.

*P*. *serotina* is a North American species that was introduced in Italy in 1922. This infesting plant is especially spreading in floodplain areas and poses a great risk to local flora due to its allelopathic strategies that change the chemical and microbial composition of the soil while also limiting the germination of seeds from local species^19^. Sexually mature plants produce an average of 6000 seeds each^26^, which are diffused mainly by frugivorous bird species^27^.

### Collection of plant material

For each species, 100 unripe and ripe fruits respectively, were sampled in natural areas of the Lombardy Po plain (Fig. 1). Specifically, *L*. *japonica* was collected in the Southern part of the Lombardy Po plain at the naturalized wet area of Cassinazza di Baselica (45°12′16.1″N, 8°36′00.7″E). *P*. *americana* was sampled in the Park of Valle Lambro (45°40′11.8″N, 9°15′23.2″E), and *P*. *serotina* was collected in the Ticino Park in the North-West Lombardy (45°40′37.9″N, 8°41′02.6″E).

Fruit from *Vaccinium myrtillus* L. were used as a standard reference for chemical analyses due to its high concentration of antioxidant compounds^28^. *V*. *myrtillus* was collected in Northern Lombardy. (45°59′00.0″N, 9°24′00.0″E).

Fruits were immediately stored at −20 °C after collection until laboratory analyses. Chemical composition and antioxidant activity analyses were performed on both unripe and ripe fruits of the three invasive plant species and of the control species as well.

### Fruit extracts

For each species and for both categories (i.e. ripe and unripe), fruits were further processed using liquid nitrogen and were powdered. A total of 3 g of material was subjected to two different extraction procedures: hydro-alcoholic and aqueous. The hydro-alcoholic procedure was performed by following the method proposed by Amigoni and co-workers^29^ without filtering with 0.45 μm PTFE filters and with a modified drug/solvent ratio: 3 g of powder were extracted using 30 mL of 70% (v/v) ethanol. The aqueous extraction was performed following the protocol described by Zhu and co-workers^30^. Both extractions for each species/fruit category were performed three times. After extraction, solvents were evaporated by a rotary evaporator and then lyophilized. Dry extracts were stored at −20 °C until analyses.

### Assessment of antioxidant activity

To evaluate the antioxidant properties of plant extracts, the DPPH assay^31^ was performed. Due to the sensitivity of this test to acid and basic pH values^32^, it was verified that the pH of extracts ranged from 4 to 8. Values obtained from unripe and ripe fruit extracts were compared with the extracts of the control species *V*. *myrtillus*. Moreover, the Folin-Ciocalteu assay^33^ was also performed to verify whether the antioxidant activity of the extracts could be related to the presence of polyphenols. Results of the DPPH and Folin-Ciocalteau assays were expressed as Trolox and gallic acid (GA) equivalents respectively, by means of calibration curves.

### Characterization of phytocomplexes

The obtained fruit extracts were characterized spectrophotometrically for the specific assessment of total flavonoids^33,34^, catechins^35^, and hydroxycinnamic acids^36^. These are colorimetric assays based on the reaction between a specific class of phytochemical and the specific reagents. A calibration curve is prepared for each assay by using increasing concentrations of a standard compound representative of the class: catechin (CAT) for flavonoids and catechins and ferulic acid (FA) for hydroxycinnamic acids. The color intensity of reacted samples was measured by a spectrophotometer and the concentration of the searched phytochemical family was calculated by means of the calibration curve. The amount of tannins was analyzed according to Porter and co-workers protocol^37^ with minor modifications; briefly, two aliquots of each sample were mixed with the reagent (50% n-butanol, 50% 12 N HCl, 0.015% FeCl_3_) and incubated for 30 min, one at room temperature and the second at 100 °C. Absorbance (Abs) was measured at 550 nm and tannin content (g/L) was calculated as (Abs_100 °C_−Abs_room temperature_) × 0.1736.

### Quantification of specific polyphenols by HPLC-DAD

Plant fruit extracts were analyzed for phenolic compounds by HPLC-DAD^38^. Samples were loaded onto a Strata-X column (33 mm polymeric sorbent 60 mg in 3 mL, Phenomenex, Torrence, CA, USA), and polyphenols were eluted by 100% v/v methanol. The eluates were completely dried in a speed vacuum at 45 °C and resuspended in 200 mL of 1:9 acetonitrile/0.2% v/v acetic acid before being directly injected into the HPLC. Polyphenols were analyzed by RP HPLC separation (column Gemini C18, 5 µm particles 250 × 4.60 mm, precolumn SecurityGuard Ea, Phenomenex, Torrence, CA, USA) equipped with an on-line diode array detector (MD-2010, Plus, Jasco Instruments, Großumstad, Germany) analyzing from 220 to 550 nm.

The adopted HPLC-DAD separation procedure allowed for the simultaneous analysis of 28 compounds among which stilbenes, phenolic acids, and flavonoids: *trans*- and *cis*-resveratrol (tRES, cRES), *trans*- and *cis*-piceid (tPIC, cPIC), *trans*- and *cis*-resveratroloside (tRDE, cRDE), piceatannol (PICEAT); gallic, protocatechuic, syringic, and vanillic acids (GA, PROTA, SIRA, VANA); caffeic, chlorogenic, *p*-coumaric, ferulic, sinapic, and *trans*-cinnamic acids (CAFA, CLORA, CUMA, FA, SINA, tCINA); catechin (CAT), epicatechin (EC), epigallocatechin 3-O-gallate (EGCG), epicatechin gallate (ECG), epigallocatechin (EGC); vanillin (VAN), naringenin (NAR), quercetin (QUERC), rutin (RUT), myricetin (MYR), and kaempferol (KAE). Based on SciFinder (https://scifinder.cas.org/), the average market price of these metabolites was assessed and used to evaluate the potential chemical value of the analyzed fruits.

### Seed viability

To estimate the invasive potential of each species through seed dispersal, the presence of mature seeds and their viability starting from unripe and ripe fruits was investigated. A total of 50 seeds per species belonging to 10 different plants were analyzed using the tetrazolium chloride test to verify the respiration of the embryos through a chemical reaction that produces a reddish stain^21^. Negative controls were performed on dead seeds obtained by heating fresh seeds to 100 °C for 1 h.

### Statistical analyses

Extraction yields data were log transformed and then analyzed by one way ANOVA with the Welch correction due to their non-homogenous variances. The Duncan test was performed to identify the samples significantly different from the others. Antioxidant activity and total phenol content of the samples were compared to those obtained from *V*. *myrtillus* extracts using the Neyman method of confidence interval at 95% level. Then to verify whether the antioxidant activity was due to the presence of polyphenols, to the degree of ripening, to the solvent of extraction, and to understand whether it could be of interest to better characterize polyphenols, we analyzed our data using linear models (LM) or, if necessary, using the generalized least squares method (GLS). Data concerning the chemical characterization of flavonoids, catechins, hydroxicinnamic acids, and tannins were analyzed as follows. Normality was verified using the Shapiro-Wilk test and homogeneity of variances was verified by Levene test. Data lacking normality were transformed by i) log transformation (flavonols), ii) square root transformation (hydroxicinnamic acids, tannins), and iii) rank transformation (catechins). One-way analysis of variance (ANOVA) was performed on transformed data (in the case of heteroscedasticity, the Welch’s correction was applied), and then data were analyzed by the Tukey-Kramer post hoc test (if the assumption of homogeneity of variances was satisfied) or by the Duncan’s multiple range test (if the assumption of homogeneity of variances was not satisfied) at a significance level of 0.05. All the analyses and graphics were produced using the Software R 1.0.44.

### Data availability

All the data supporting the results presented here have been provided within the manuscript’s tables or as Supplementary material.

## Results

### Yields of extraction and antioxidant activity

**Figure 2:**
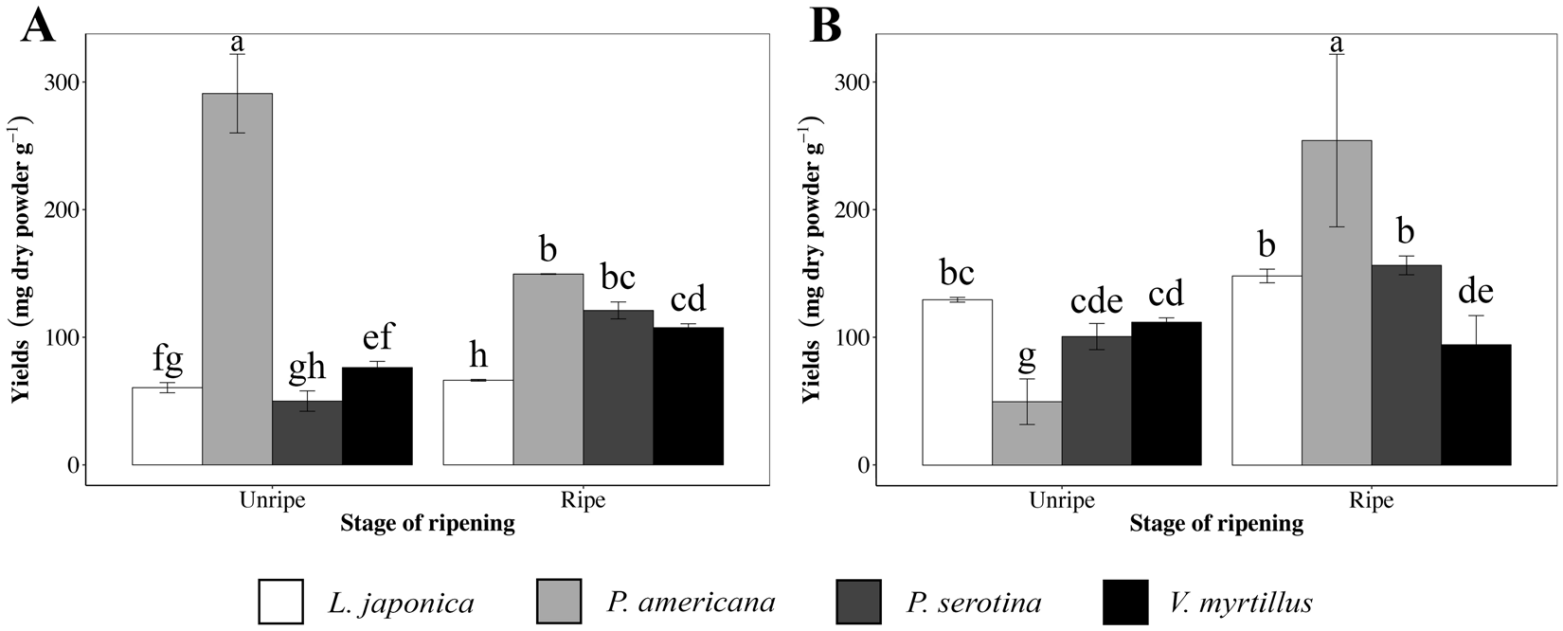
Yields of the hydro-alcoholic (**A**) and aqueous (**B**) extractions conducted on fruits from the three invasive plant species and the control species *V*. *myrtillus*. Data are reported as mg of dry powder obtained from 1 g of fresh fruit and expressed as the mean ± standard deviation (SD). The letters above each bar indicate the results of Duncan’s multiple range test (p < 0.05). Average values with standard deviation followed by the same letters are not significantly different.

Figure 2 provides a brief comparison of extraction yields for all the investigated species and tested protocols. *P*. *americana* showed the highest yield of extraction in all cases with the only exception of unripe fruits extracted with water. *L*. *japonica* showed a statistically lower yield of extraction than the control *V*. *myrtillus* in the hydro-alcoholic condition, and the opposite situation occurred with the aqueous extraction protocol. Finally, *P*. *serotina* extraction yields starting from unripe fruits were lower than in the ripe ones, even though yields of aqueous extraction were comparable to that of *V*. *myrtillus*.

**Figure 3:**
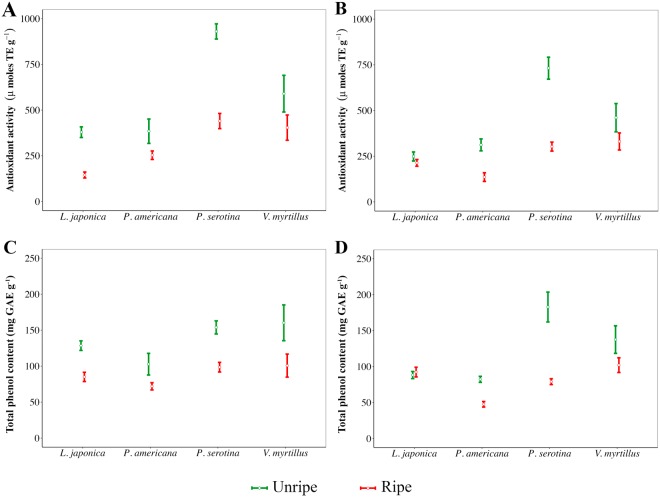
Results of DPPH (**A**,**B**) and Folin-Ciocalteu assays (**C**,**D**). Results are showed for the hydro-alcoholic extraction (**A**,**C**) and the aqueous one (**B**,**D**) conducted on fruits of the three investigated invasive plants and the control species *V*. *myrtillus*. Green bars refer to the unripe fruits, whereas red bars indicate ripe ones. White dots express the mean values and the bars represent the 95% confidence interval. TE: Trolox Equivalents; GAE: Gallic Acid Equivalents.

Antioxidant activity values estimated by the DPPH assay are shown in Fig. 3(A,B). The highest value was observed for *P*. *serotina* unripe fruit, where both hydro-alcoholic and aqueous extracts showed to be 1.6 times more active than *V*. *myrtillus* unripe extracts. When fruits ripen, the statistical difference between the antioxidant activity of *P*. *serotina* extracts and that of the control species disappears, and ripe *P*. *serotina* fruits are generally two times less active that unripe ones. *Phytolacca americana* and *L*. *japonica* showed values of antioxidant activity quite lower than *V*. *myrtillus*, and these species also show a higher antioxidant activity in unripe conditions.

The total phenol content assessed through Folin-Ciocalteu assay is reported in Fig. 3(C,D). Generally, unripe fruits are up to 2.3 times richer in polyphenols than the ripe ones (i.e. *P*. *serotina* aqueous extracts). Although *L*. *japonica* and *P*. *americana* extracts were not as rich in polyphenols as *V*. *myrtillus*, the hydro-alcoholic extraction enhanced their quantity, especially in the case of the latter species.

To evaluate the possible elements influencing antioxidant activity, we adopted a regression analysis to assess the effect of biological (ripening) and chemical (polyphenols and solvent of extraction) variables (Table 1). The occurrence of polyphenols is one of the most significant factors inducing antioxidant activity (p < 0.001) in all the investigated plants. In *P*. *serotina* and *P*. *americana*, unripeness plays a relevant role in the antioxidant activity (*P*. *serotina* p < 0.001, and *P*. *americana* p = 0.052). In the case of *P*. *serotina*, the antioxidant activity also depends on the interaction between the amount of polyphenols and the method of extraction (p < 0.001). This condition can be explained by taking into account the different extraction capacity and specificity of hydro-alcoholic solvent and water. For example, it was previously reported that 95% (v/v) ethanol is able to extract a larger amount and spectrum of phenolic compounds from red and white grape pomace with respect to water, and alcoholic extract possesses higher antioxidant activity^39,40^.

**Table 1:** Summary of regression analyses to assess biological and chemical variables influencing the antioxidant activity of invasive plant species extracts.

Species	Best model	Chemical variables		Biological variables	Interaction between variables	
		Polyphenols	Solvent	Ripening	Polyphenols*Ripening	Polyphenols*Solvent
*L*. *japonica*	GLS, ΔAICc = 9.27	Value: 2.86; t-value: 5.42; P < 0.001	Value: 176.17; t-value: 1.85; P = 0.0715	—	Value: −0.79; t-value: −2.92; P = 0.0056	—
*P*. *americana*	LM, Adj. *R*^2^ = 0.8622, *F*_4,41_ = 71.41, *P* < 0.001, ΔAICc = 0.27	Value: 2.97; t-value: 5.38; P < 0.001	—	Value: −113.20, t-value: −2.00; P = 0.052	Value: 1.04; t-value: 1.44; P = 0.158	Value: −0.27; t-value: −1.45; P = 0.154
*P*. *serotina*	GLS, ΔAICc = 7.81	Value: 2.83; t-value: 6.96; P < 0.001	—	Value: −290.18; t-value: −8.35; P < 0.001	—	Value: −1.29; t-value: −9.99; P < 0.001

For each investigated species, the best fitting model was selected by evaluation of the ΔAICc. Empty fields indicate that the variable considered was not significant and detrimental to the model.

For the antioxidant properties of *L*. *japonica*, the interaction between the amount of polyphenols and the degree of ripening also appears to be significant, meaning that unripe fruit contain a higher amount of polyphenols than the ripe ones (p = 0.006).

### Chemical characterization and identification of high-added values compounds

Given the well-recognized antioxidant activity of polyphenols, the occurrence of the most important classes was spectrophotometrically evaluated (Table 2). Results showed that aqueous extracts of unripe fruit from *P*. *serotina* had a higher amount of flavonoids compared to the ripe ones (up to 7.41 times) including catechins (not detected in ripe fruits), tannins (up to 5.5 times), and hydroxycinnamic acids (up to 4.1 times). A similar pattern was observed in hydro-alcoholic conditions. Noteworthy, the amount of hydroxycinnamic acids *in P*. *serotina* reached an average value of 650 mg of ferulic acid equivalents (FA eq.) per g of dry extracts in the aqueous extract from unripe fruits (more than two times the amount of FA eq. in *V*. *myrtillus*). Also, hydro-alcoholic extracts of unripe *L*. *japonica* fruits exhibited high amounts of hydroxycinnamic acids (386.68 mg of FA eq. per gram of dry extract). The highest amount of catechins was found in the aqueous extracts of *P*. *serotina* (average value of 73.69 mg of catechin equivalents per gram of dry extract), whereas no catechins were found in extracts from *L*. *japonica* and *P*. *americana* fruits.

**Table 2:** Characterization of the main important classes of polyphenols by spectrophotometric analyses.

Species	Ripening	Extraction	Flavonoids (mg CAT eq *g^−1^)	Catechins (mg CAT eq *g^−1^)	Hydroxicinnamic acids (mg FA eq *g^−1^)	Tannins (mg*g^−1^)
*L*. *japonica*	unripe	HA	34.14 ± 0.18	Undet.	386.78 ± 15.08	38.37 ± 2.27
		A	19.69 ± 0.27	Undet.	117.05 ± 5.36	21.40 ± 1.61
	ripe	HA	12.37 ± 0.48	Undet.	52.06 ± 4.66	13.02 ± 0.43
		A	16.06 ± 0.45	Undet.	166.41 ± 52.21	18.49 ± 0.52
*P*. *americana*	unripe	HA	36.54 ± 0.36	Undet.	44.76 ± 4.67	12.85 ± 1.90
		A	6.43 ± 0.04	Undet.	7.06 ± 2.32	4.65 ± 0.00
	ripe	HA	27.50 ± 5.09	Undet.	17.00 ± 0.00	Undet.
		A	3.65 ± 0.00	Undet.	3.68 ± 0.42	Undet.
*P*. *serotina*	unripe	HA	53.10 ± 0.14	16.39 ± 0.62	374.08 ± 31.74	82.15 ± 1.35
		A	72.90 ± 2.97	73.69 ± 9.79	650.60 ± 62.84	96.15 ± 0.55
	ripe	HA	10.18 ± 1.59	Undet.	200.84 ± 24.19	21.20 ± 1.85
		A	9.83 ± 1.27	Undet.	159.33 ± 18.41	17.45 ± 0.20
*V*. *myrtillus*	unripe	HA	32.75 ± 0.78	20.21 ± 1.95	252.67 ± 19.33	69.25 ± 2.60
		A	26.10 ± 0.14	4.42 ± 0.26	263.05 ± 20.35	39.80 ± 0.55
	ripe	HA	7.79 ± 2.82	Undet.	68.02 ± 18.01	25.30 ± 3.35
		A	5.54 ± 0.91	Undet.	46.68 ± 4.18	19.35 ± 0.40

Hydro-alcoholic (HA) and aqueous (A) extracts of *L. japonica, P. americana, P. serotina* and the reference species *V. myrtillus*. Data are expressed as mg of catechin (CAT) and mg of ferulic acid (FA) equivalents per gram of lyophilized extract for flavonoids (including catechins) and hydroxicinnamic acids respectively, and as mg of detected compounds per g of dry extract for tannins. Results are the mean of three replicates ± Standard Deviation (SD).

All the unripe fruits processed with a hydro-alcoholic extraction showed similar or higher amounts of flavonoids than *V*. *myrtillus*. However, aqueous extracts of unripe *P*. *serotina* fruit showed flavonoid and tannin contents 2.8 and 2.4 times higher than *V*. *myrtillus* in the same conditions.

To better understand the biochemical profile of extracts obtained from the studied species, a total of 28 standard high-added value compounds were investigated by HPLC-DAD (Supplementary Table 1). Noteworthy, the most interesting compounds found in unripe fruit from *P*. *serotina* were chlorogenic acid (CLORA, avg. 3.137 mg*g^−1^) and rutin (RUT, avg. 1.856 mg*g^−1^ in hydro-alcoholic extract and 1.938 mg*g^−1^ in the aqueous one). In the same samples, catechins (e.g., epigallocatechin 3-O-gallate EGCG) were also detected (avg. 0.180 mg*g^−1^ in aqueous extract). A large amount of the unidentified chromatographic peaks showed an absorbance spectrum characteristic of catechins, thus confirming that these compounds are present in significant amounts. Finally, *P*. *serotina* unripe fruit showed some glycosylated stilbenes, particularly *trans*-piceid (tPIC avg. 0.596 mg * g^−1^).

RUT and some catechins were also detected in unripe fruits of *P*. *americana*, especially in the hydro-alcoholic extracts (RUT avg. 1.885 mg*g^−1^).

The most abundant metabolite found in *L*. *japonica* unripe fruits was caffeic acid (CAFA avg. 7.052 mg*g^−1^ in hydro-alcoholic extracts). In addition, some flavonoids such as RUT (avg. 3.350 mg*g^−1^) and quercetin (QUERC avg. 0.672 mg*g^−1^) were also detected in hydro-alcoholic extracts from unripe fruits.

### Invasion risk and values of alien plant fruits

To better evaluate the risk of invasion in the three investigated plants, the viability of 100 seeds of each species was assessed (Supplementary Figure 1). Only seeds belonging to ripe fruits were analyzed, because unripe ones had no or contained only undeveloped seeds. The obtained results suggested that 54% of *L*. *japonica*, 56% of *P*. *americana*, and 72% of *P*. *serotina* were viable and therefore potentially active in enhancing the invasiveness of the sampled populations (Table 3). To estimate the chemical and biological value of these fruits/seeds for human aims, the yield of extraction for each species was also reported. Starting from 100 fruit we obtained a minimum of 0.749 g of dry extract (ripe fruits, hydro-alcoholic extraction) from *L*. *japonica* to a maximum of 7.069 g of dry extract (unripe fruits, aqueous extraction) from *P*. *americana*. Considering the best yield of extraction, the aqueous procedure obtained 7.043 mg of CLORA, 4.277 mg of RUT, and almost 0.400 mg of EGCG in *P*. *serotina*. Based on a rough estimate conducted by exploring the SciFinder database, these extracted chemicals could have a commercial value ranging from € 7.77 with the hydro-alcoholic procedure to € 58.97 with the aqueous one. In the case of *P*. *americana*, 100 unripe fruits extracted with the hydro-alcoholic solution produced about 8.010 mg of RUT and 2.418 mg of CAT with a total commercial value of € 10.89. Finally, *L*. *japonica* yielded 6.227 mg of CAFA from the hydro-alcoholic extraction of unripe fruits and almost 3 mg of RUT as well as 0.595 of QUERC in the same conditions with a total commercial value of € 13.45. In some cases, ripe fruit also showed a conspicuous economic value (e.g., aqueous extracts of *P*. *americana* and hydro-alcoholic extracts of *P*. *serotina* reached an estimated value of € 56.13 and of € 77.12 respectively). However, antioxidant activity detected by the DPPH assay was higher in unripe conditions than ripe ones.

**Table 3:** Seed viability and the bioprospecting value of the three invasive plant species.

Species	Ripening	Viability	Extraction	Yields dry extract (g) * 100 fruits^−1^	Valuable compounds (mg*100 fruits^−1^)	Market price (€/mg)	Value (€) * 100 fruits
*L*. *japonica*	unripe	—	HA	0.883	**CAFA:** 6.227;	2.066	12.863
					**RUT:** 2.958;	0.0026	0.008
					**QUERC:** 0.593	0.970	0.575
			A	1.842	**CAFA:** 1.509;	2.066	3.117
					**RUT:** 1.319;	0.0026	0.003
					**QUERC:** 0.357	0.970	0.346
	ripe	54%	HA	0.749	**CAFA:** 1.183;	2.066	2.444
					**RUT**: 1.085;	0.0026	0.003
					**QUERC:** 0.354	0.970	0.344
			A	1.631	**CAFA:** 1.295;	2.066	2.675
					**RUT:** 1.235;	0.0026	0.003
					**QUERC:** 0.667	0.970	0.647
*P*. *americana*	unripe	—	HA	4.249	**RUT:** 8.010;	0.0026	0.020
					**CAT:** 2.418	4.496	10.872
			A	7.069	**RUT:** 0.283	0.0026	0.001
	ripe	56%	HA	2.931	**RUT:** 3.379;	0.0026	0.009
					**EGC:** 2.292	16.790	38.480
			A	4.853	**EGC:** 3.343	16.790	56.130
*P*. *serotina*	unripe	—	HA	1.125	**CLORA:** 3.529;	0.16	0.565
					**RUT:** 2.088;	0.0026	0.052
					**tPIC:** 0.671;	0.54	0.360
					**EGCG:** 0.1	68	6.800
			A	2.207	**CLORA:** 7.043;	0.160	1.130
					**RUT:** 4.277;	0.0026	0.111
					**EGC**: 1.83	16.790	30.730
					**EGCG:** 0.397	68	27.000
	ripe	72%	HA	3.135	**CLORA**: 1.919;	0.16	0.307
					**RUT**: 1.452;	0.0026	0.004
					**EGC**: 4.677	16.790	77.120
			A	3.429	**CLORA**: 2.438;	0.16	0.390
					**RUT:** 0.772;	0.0026	0.002

For each species, the percentage of viable seeds assessed by the tetrazolium-chloride test is reported. Yields of extraction and the quantification of high-valuable compounds for each ripening stage and protocol of extraction are provided. Moreover, the market price of each metabolite was reported. The number of fruits considered = 100.

## Discussion

In this work we propose the exploitation of invasive plant species in a framework of bioprospecting^41^ to obtain high-added value compounds or phytocomplexes. Fruit collection for phytochemical extraction could also be seen as a way to prevent seed dispersal and therefore limit plant invasiveness.

Our results suggest that fruits from the investigated plants contain compounds with beneficial effects on human health that also have a high benchmark value on the market. This is the case of rutin, which is commonly known as a natural anti-inflammatory and antidiabetic compound^42^, stilbenes (*trans-*piceid and picetannol) known for their antimicrobial, anti-inflammatory, and antitumoral interest^43^, and hydroxycinnamic acids, such as chlorogenic and caffeic acids for which beneficial properties have been recognized as well^44^. Moreover, some of these metabolites show a high stability during gastric digestion, as is the case of piceid and rutin, which are glycolsylated and therefore protected during gastric digestion^45^. These two molecules are converted in the gut into the corresponding aglycones resveratrol and quercetin, which are polyphenols displaying a variety of biological activities in humans^46,47^ and are used as active components of several food supplements commercially available. This condition enhances the potential nutraceutical value of these extracts and supports their exploitation through bioprospecting.

We are aware that our proposal, if misunderstood, could open the possibility for the spread of invasive plants or even stimulate their deliberate cultivation. At the same time, we underline that the evolutionary history of plants shows that anthropogenic stress and overexploitation (including biopiracy) could result in a reliable containment or extinction of plant populations^48^. A typical example is *Taxus contorta* L., a Pleistocene tree on the brink of extinction in several Middle East regions, because bark and needles are harvested to produce anti-cancer medicines^49^. A more dramatic case is that of Coco de Mer (*Lodoicea maldivica* (J.F.Gmel.) Pers.) from the Seychelles, whose fruits are overexploited due to their supposed, but not scientifically confirmed, aphrodisiac properties^50^. Also plants having a wider geographical distribution, such as the European mountain species *Arnica montana* L.^51^ and *Gentiana lutea* L.^52^ have being interested by overexploitation and consequent extinction risk due to a growing market demand.

Given these considerations, we hypothesize that the harvest of invasive plants for bioprospecting purposes could help limit their uncontrolled spread in European and other countries opening new scenarios in the field of biodiversity conservation.

In Europe, the cost to manage invasive plants is estimated at 12 billion Euros per year^53^, in the USA, it is about 35 billion of dollars^54^, and in Australia, it is in about 4 billion dollars^15^. Despite these economic investments, the eradication effectiveness is limited to spot areas and the dissemination of invasive species mediated by natural ecosystem services is still advancing^20,55^. In the Lombardy Po plain, containment activities that have been conducted on the species investigated in this study are not effective. For example, in the case of *P*. *serotina*, an intensive management action was started in 1997 in the Ticino valley Park to try to limit the species. After 11 years, and despite costing 830000 Euros to contain it, this species enhanced its diffusion, overcoming the boundaries of the park^56^. Moreover, management activities produced indirect costs, such as those related to the waste disposal of the biomasses derived by pruning and extirpation actions. In this context, bioprospecting on plant biomass could help solve not only the problem of spreading invasive species, but also the management of waste. This agrees with current guidelines of agro-bioeconomy (see UE n.1141/2014), that stimulates the development of circular solutions that are able to transform waste into resources^57^.

An important element is the selection of the most suitable biomass. This study was focused on fruits, and our analysis suggested that most chemical compounds (e.g. polyphenols) and biological activities occur in unripe fruits rather than in ripe ones.

This is really relevant, because collecting fruit before they reach the ‘invasive developmental stage’ prevents seed dispersal. Moreover, we would like to stress that the Po plain is an important bird stopover area, and in a previous study, it was demonstrated that local and migratory birds are particularly attracted to dark fruits, such as *L*. *japonica* and *P*. *americana*^20^. Many migratory species prefer these fruits over those of local species, such as *Viburnum* spp. and *Sambucus* spp. Only after eradication actions of *L*. *japonica* and the massive restoration of local species did the diet of birds changed towards a greater exploitation of indigenous plant species^20^. Thus, at the local scale, bioprospecting activities on invasive species could enhance the success of ecological restoration plans.

From a technical point of view, the harvesting of unripe fruits could sound expensive and not sustainable. However, this activity could fit with a circular economy strategy for biodiversity management where ‘plant pickers’ are directly involved by forest and territory authorities to collect high-added value plant biomasses destined to processing by phytochemical companies. We underline that many bioactive metabolites and food ingredients are still today obtained from spontaneous or proto-domestic species and harvested by pickers^58^. Mediterranean diet is recently facing a comeback to the collection and use of spontaneous herbs such as the wild asparagus (*Asparagus acutifolius* L.) that is rich in antioxidants, wild cabbages (*Brassica* spp.) that contains high levels of folates and the maidenstears (*Silene vulgaris* (Moench) Garcke) that shows important amount of Omega-3 fatty acid^59,60^. Many companies involved in drugs and cosmetics production, regularly purchase great batches of wild plants to exploit their well-known bioactive components, including poisonous species as well. This is the case of *Atropa belladonna* L. that is already used to extract analgesic and antispasmodic phytocomplexes. Also fruit belonging to *V*. *myrtillus*, our experimental control species, are harvested at high altitudes on the Alps and Appennins^61^ (where they grow spontaneously), and other *Vaccinium* sp. are still harvested in nature in Canada and in the USA. The cultivated species *V*. *corymbosum* L. is polyploid, and although it has a higher production yield, is not as rich in antioxidants and anthocyanins as *V*. *myrtillus*^62^. The occurrence of secondary metabolites is also related to the response of the plant to different environmental conditions. Some studies highlighted how invasive plants show an altered metabolic profile in terms of secondary compounds (e.g. polyphenols) when they colonize new non-native territories^63,64^. An allelopathic response could explain this phenomenon: for example, caffeic acid extracted from *L*. *japonica* fruits is a typical allelopathic compound^65^. Moreover, it is known that also flowers and buds of this species are particularly rich in valuable compounds, such as chlorogenic acid and luteoloside that contribute to increase the economic value of *L*. *japonica* in terms of secondary metabolites^66,67^. Modifications in plant phytocomplexes during invasions also occur in *P*. *serotina* drupes. These fruits are appreciated by humans in native territories (USA and Mexico) where they are eaten fresh or utilized to prepare jams^68^, while in European countries they are poorly consumed due to bitter taste.

In this study, the chemical composition of the three alien species in their native areas was not assessed; however, a comparative analysis of secondary compounds from the original area vs. those in invaded areas could permit the identification of interesting metabolites for human aims and the evaluation of factors enhancing their presence. This could represent a core aspect of a bioprospecting approach along with the study of metabolic pathways and genes involved in the biosynthesis of high-added value compounds and the role played by the environment^69^.

In a conservation context, the characterization of allelopathic metabolites and of plant invasiveness must be performed before the import of an alien species in order to prevent its uncontrolled diffusion as has happened in the cases of *L*. *japonica* and *P*. *serotina*. The latter was introduced in Europe due to the high wood quality in its native territories; however, once established in Po plain, its xilema developed enormous tracheas making it rotten and of low interest for wood industries^19^. Therefore, this plague was introduced without any economic advantage.

In conclusion, a bioprospecting approach focused on alien species represents a promising strategy for discovering new metabolites, as well as to contain, or at least plan prevention strategies against plant invasiveness. This kind of approach could also support a sort of circular economy in the framework of invasive species management procedures, meaning that the metabolites found in the extracted phytocomplexes could produce economic gains that can be used to support, at least partially, the costs required for eradication actions.

Nowadays, there is an increasing trend to integrate ecosystem service topics within the management plans and strategies of protected areas. In this context, we encourage the adoption of bioprospecting focused especially on invasive species as an integrative element of these services to economically support (e.g. involving phytochemical industry) local biodiversity.

## Electronic supplementary material

Fig. S1 and Table S1
