# Publisher Correction: Bioprospecting on invasive plant species to prevent seed dispersal

**DOI:** 10.1038/s41598-018-29096-0

**Published:** 2018-07-19

**Authors:** Lorenzo Guzzetti, Andrea Galimberti, Ilaria Bruni, Chiara Magoni, Maura Ferri, Annalisa Tassoni, Enrico Sangiovanni, Mario Dell’Agli, Massimo Labra

**Affiliations:** 1 Zooplantlab, Department of Biotechnology and Biosciences, University of Milano-Bicocca, Piazza della Scienza 2, I-20126 Milano, Italy; 2 Department of Biological, Geological and Environmental Sciences, University of Bologna, via Irnerio 42, 40126 Bologna, Italy; 3 Department of Civil, Chemical, Environmental and Materials Engineering, University of Bologna, via Terracini 28, 40131 Bologna, Italy; 4 Laboratory of Pharmacognosy, Department of Pharmacological and Biomolecular Sciences, Università degli Studi di Milano, Via Balzaretti 9, 20133 Milano, Italy

Correction to: *Scientific Reports* 10.1038/s41598-017-14183-5, published online 23 October 2017

The original version of this Article contained a typographical error in the spelling of the author Mario Dell’Agli, which was incorrectly given as Mario Dell’ Agli. This has now been corrected in the PDF and HTML versions of the Articles.

