# Supplementary material for: Bioprospecting on invasive plant species to prevent seed dispersal
Source: Sci Rep. 2017 Oct 23;7:13799. doi: 10.1038/s41598-017-14183-5 (PMC5653781; doi:10.1038/s41598-017-14183-5)
Supplement: Supplementary file 1 — Fig. S1 and Table S1 [file 41598_2017_14183_MOESM1_ESM.pdf]

Bioprospecting on invasive plant species to prevent seed dispersal

Lorenzo Guzzetti[a], Andrea Galimberti[a], Ilaria Bruni[a], Chiara Magoni[a], Maura Ferri[b,c], Annalisa Tassoni[b], Enrico Sangiovanni[d], Mario Dell'Agli[d], Massimo Labra[a*]

Fig. S1 Images of fruits and seeds of the three invasive plant species *L. japonica* (A, D), *P. americana* (B, E), and *P. serotina* (C, F). Seed viability was analyzed with the Tetrazolium Chloride viability test where red staining corresponds to active cellular respiration.

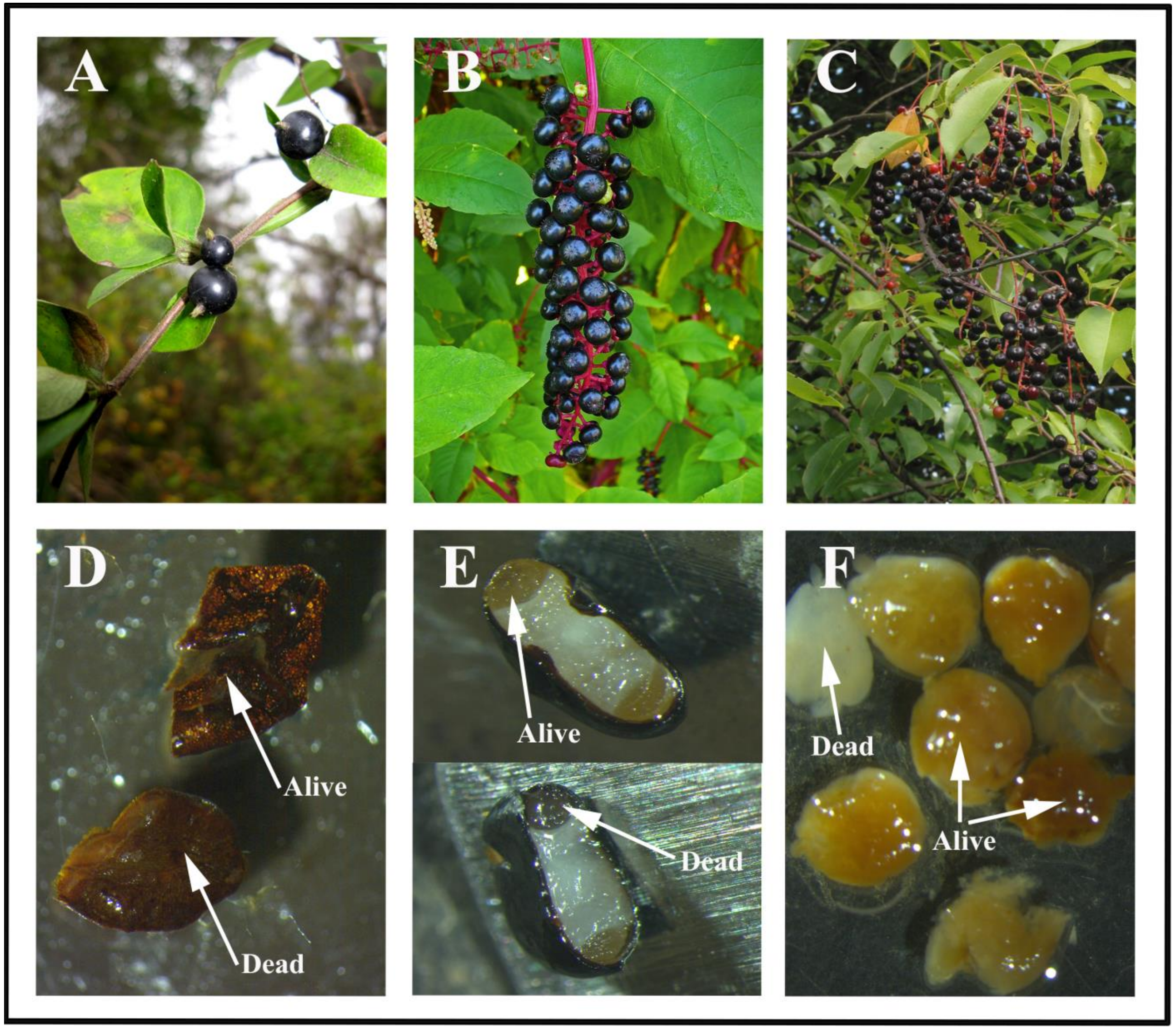

Table S1 Quantification of specific polyphenols by HPLC-DAD in the hydro-alcoholic (HA) and aqueous (A) extracts of *L. japonica*, *P. americana*, *P. serotina*, and *V. myrtillus*. Results are expressed as mg of compound/g dry extract ± SD.

Plant	Ripening	Extraction	Hydroxibenzoic acids	Hydroxicinnamic acids	Flavonoids	Flavan-3-ols	Stilbenes	Others
L. japonica	unripe	Hydro-alcoholic		**CAFA**: 7.052 ± 0.299	**RUT:** 3.350 ± 0.103; **QUERC:** 0.672 ± 0.141	**EC:** 0.600 ± 0.091		
		Aqueous	**PROTA:** 0.064 ± 0.001	**CAFA**: 0.819 ± 0.051; **CUMA:** 0.062 ± 0.018	**RUT:** 0.716 ± 0.027; **QUERC:** 0.194 ± 0.024	**EC:** 0.323 ± 0.091		
	ripe	Hydro-alcoholic		**CAFA**: 1.579 ± 0.016	**RUT:** 1.449 ± 0.105; **QUERC:** 0.473 ± 0.074			
		Aqueous		**CAFA**: 0.794 ± 0.013 **CUMA:** 0.050 ± 0.006	**RUT:** 0.757 ± 0.014; **QUERC:** 0.409 ± 0.056			
P. americana	unripe	Hydro-alcoholic	**SIRA:** 0.035 ± 0.017	**CAFA:** 0.015 ± 0.004; **CUMA:** 0.021 ± 0.009	**RUT:** 1.885 ± 0.773	**CAT:** 0.569± 0.226; **EGC:** 0.126 ± 0.121		
		Aqueous	**SIRA:** 0.018 ± 0.003	**CAFA:** 0.010 ± 0.001; **CUMA:** 0.036 ± 0.007	**RUT:** 0.040 ± 0.007			
	ripe	Hydro-alcoholic	**SIRA:** 0.031 ± 0.002		**RUT:** 1.153 ± 0.008	**EGC:** 0.782 ± 0.012	**cPIC:** 0.156 ± 0.06	**cRDE**: 0.080 ± 0.024
		Aqueous	**SIRA:** 0.027 ± 0.005		**RUT:** 0.056 ± 0.008	**EGC:** 0.689 ± 0.003	**cPIC:** 0.032 ± 0.003	**cRDE**: 0.018 ± 0.002
P. serotina	unripe	Hydro-alcoholic		**CLORA**: 3.137 ± 0.149; **CAFA**: 0.466 ± 0.021	**RUT:** 1.856 ± 0.036	**EC:** 2.409 ± 0.077; **EGCG:** 0.089 ± 0.070	**tPIC**: 0.596 ± 0.011; **PICEAT1:** 0.31 ± 0.016; **PICEAT2:** 0.332 ± 0.002	
		Aqueous		**CLORA**: 3.191 ± 0.078	**RUT:** 1.938 ± 0.023	**CAT:** 0.836 ± 0.047; **EC:** 2.552 ± 0.372; **EGC:** 0.831 ± 0.157; **EGCG:** 0.180 ± 0.009	**PICEAT1:** 0.324 ± 0.032; **PICEAT2:** 0.391 ± 0.032	
	ripe	Hydro-alcoholic	**PROTA:** 0.419 ± 0.077	**CLORA**: 0.612 ± 0.036	**RUT:** 0.463 ± 0.032	**EC:** 0.415 ± 0.2; **EGC:** 1.492 ±		

						0.096		
		Aqueous	**PROTA:** 0.261 ± 0.031	**CLORA**: 0.711 ± 0.057	**RUT:** 0.225 ± 0.013	**EC:** 2.552 ± 0.372		**VAN:** 0.162 ± 0.007
V. myrtillus	unripe	Hydro-alcoholic		**CAFA**: 0.165 ± 0.007; **CLORA**: 6.352 ± 1.113; **CUMA:** 0.201 ± 0.010		**EC:** 1.918 ± 0.054; **EGC:** 0.364 ± 0.019	**tRDE1:** 1.013 ± 0.108; **tRDE2:** 10.398 ± 1.941	
		Aqueous	**PROTA:** 0.217 ± 0.006	**CAFA**: 0,166 ± 0,023; **CLORA**: 5.67 ± 0.387; **CUMA:** 0.386 ± 0.044		**CAT:** 0.169; **EC:** 1.12 ± 0.108; **EGCG:** 0.200 ± 0.084	**tRDE1:** 0.732 ± 0.047; **tRDE2:** 7.479 ± 0.186	
	ripe	Hydro-alcoholic		**CLORA**: 1.325 ± 0.083; **CUMA:** 0.08 ± 0.003		**CAT:** 0.274 ± 0.055; **EC:** 0.431 ± 0.129	**tRDE1:** 0.57 ± 0.056; **tRDE2:** 4.87 ± 0.068	
		Aqueous	**GA:** 0.314 ± 0.028; **PROTA:** 0.266 ± 0.004	**CLORA**: 0.788 ± 0.092		**EGC:** 0.293 ± 0.042	**tRDE1:** 0.532 ± 0.003; **tRDE2:** 3.597 ± 0.033	
